# Aging, Senescent Cells, and Senolytic Drugs: The Path to Translation

**DOI:** 10.1093/geroni/igaa057.3198

**Published:** 2020-12-16

**Authors:** James Kirkland

**Affiliations:** Mayo Clinic, Rochester, Minnesota, United States

## Abstract

Senescent cells (SC) accumulate with aging and at causal sites of multiple chronic disorders and diseases, including those accounting for the bulk of morbidity, mortality, and health expenditures. SC do not replicate. Some SC release factors that cause tissue dysfunction, the senescence-associated secretory phenotype (SASP). Transplanting small numbers of SC into younger mice to above a critical threshold leads to frailty, early onset of multiple age-related diseases, and premature death. A report in 2004 showing caloric restriction causes both healthspan extension and delayed SC accumulation prompted us to begin efforts to discover senolytic drugs, agents that selectively eliminate SC. We used a hypothesis-driven, mechanism-based strategy to discover senolytics, reasoning that senescent cell anti-apoptotic pathways (SCAPs) exist that defend SC against their own SASP, allowing them to survive, despite killing neighboring cells. Senolytics cause SC apoptosis by transiently disabling these SCAPs. Because SC take weeks to re-accumulate, senolytics can be administered intermittently – a “hit-and-run” approach. Senolytics delay, prevent, or alleviate frailty and cardiovascular, neuropsychiatric, liver, kidney, musculoskeletal, lung, eye, hematological, metabolic, and skin disorders as well as complications of organ transplantation, radiation, and cancer treatment in pre-clinical models. As anticipated for agents targeting the fundamental aging mechanisms that are “root cause” contributors to multiple disorders, potential uses of senolytics are protean, potentially alleviating over 40 conditions in preclinical studies, opening a new route for treating age-related dysfunction and diseases. We review the discovery of senolytics and potential strategies for translation into the clinic. Early trials indicate effectiveness of senolytics in humans.

